# Supplementary material for: Monocyte Subsets in Patients with Chronic Heart Failure Treated with Cardiac Resynchronization Therapy
Source: Cells. 2021 Dec 9;10(12):3482. doi: 10.3390/cells10123482 (PMC8700101; doi:10.3390/cells10123482)
Supplement: Supplementary file 1 [file cells-10-03482-s001.zip › cells-1468332-supplementary.pdf]

Supplementary materials

Table S1. Correlation of monocyte CD14^{+}CD16^{++} non-classical, CD14^{++}CD16^{+} intermediate, and CD14^{++}CD16^{-} classical monocytes subsets with selected biochemical parameters in patients with heart failure with reduced ejection fraction (HFrEF).

	**Non-classical monocytes CD14+CD16++**	**Intermediate monocytes CD14++CD16+**	**Classical monocytes CD14++CD16-**	**Transferrin saturation**	**IP-10**	**IL-31**	**BNP**	**LVDD**	**TWEAK**	**TSLP**	**Total cholesterol**	**Iron**	**MDC**	**Peak VO2**	**LVEF**
**Non-classical monocytes CD14+CD16++**	1		-												
**Intermediate monocytes CD14++CD16+**	0.242	1													
**Classical monocytes CD14++CD16-**	-0.592	-0.441	1												
**Transferrin saturation**	0.145	0.042	-0.343	1											
**IP-10**	0.372	0.140	-0.129	0.005	1										
**IL-31**	-0.411	-0.172	0.403	-0.302	-0.015	1									

**BNP**	0.090	0.016	-0.126	-0.007	-0.393	0.019	1								
**LVEDD**	0.003	0.233	-0.125	-0.066	-0.164	-0.168	0.246	1							
**TWEAK**	-0.235	-0.167	0.314	0.151	-0.261	0.067	-0.030	-0.183	1						
**TSLP**	-0.458	-0.382	0.620	-0.207	-0.005	0.791	-0.106	-0.172	-0.055	1					
**Total cholesterol**	0.180	0.157	-0.320	0.201	-0.025	-0.290	-0.024	0.033	-0.192	-0.250	1				
**Iron**	0.138	0.125	-0.370	0.831	0.023	-0.304	-0.068	-0.050	0.269	-0.344	0.335	1			
**MDC**	0.219	0.165	-0.405	0.053	-0.197	-0.136	0.202	0.303	-0.015	-0.495	0.024	0.143	1		
**Peak VO2**	-0.123	0.037	0.040	0.016	0.231	-0.025	-0.306	0.137	0.110	-0.007	0.100	0.037	0.029	1	
**LVEF**	-0.109	0.065	0.009	-0.004	0.153	0.036	-0.316	-0.326	0.236	-0.267	0.005	0.020	-0.119	0.343	1

Abbreviations: BNP, brain natriuretic peptide; IP-10, interferon gamma-induced protein 10; IL-31, interleukin 31; LVEDD, left ventricular end-diastolic diameter; LVEF, left ventricular ejection fraction; MDC, macrophage-derived chemokine; peak VO2, peak oxygen uptake; sTWEAK, soluble tumor necrosis factor-like weak inducer of apoptosis; TSLP, thymic stromal lymphopoietin

Data are presented as R – correlation coefficient value. Statistically significant parameters R – correlation coefficient for $p<0.05$ are marked in red.
